# Terahertz nanofuse by a single nanowire-combined nanoantenna

**DOI:** 10.1515/nanoph-2022-0768

**Published:** 2023-03-08

**Authors:** Geunchang Choi, Yeeun Roh, Minah Seo

**Affiliations:** School of Electrical and Electronics Engineering, Chung-Ang University, Seoul, 06974, Republic of Korea; Sensor System Research Center, Korea Institute of Science and Technology (KIST), Seoul, 02792, Republic of Korea; KU-KIST Graduate School of Converging Science and Technology, Korea University, Seoul, 02841, Republic of Korea

**Keywords:** nanofuse system, nanowire, terahertz metamaterials

## Abstract

We propose a terahertz nanofuse through irreversible modulations in transmitted terahertz using nanowires-combined nanoantenna structures. Semiconductor and metal nanowires show irreversible reconfiguration in their geometry at an incident field of 20 kV/cm. The concept can be explained by terahertz-field-induced ionization or electromigration. A strongly localized field due to geometrical conditions causes a floated metal nanowire from one side of a metal nanoantenna to touch the opposite side, bridging two separate metal plates and creating a junction. For the bridged nanoantenna, the highly enhanced field induced the breaking of the connection across the metal sides of a nanoantenna. In the bridging and breaking cases, permanent transformation occurs in opposite structural forms. It encompasses a potential application as an optical fuse to protect sensitive terahertz devices under excessive field focus.

## 1 Introduction

Recently, terahertz (THz) applications with metamaterials are gaining attention, and they can potentially be used to increase detection sensitivity through strong localization and enhancement of the incident THz electromagnetic field [1–5]. Using field confinement of metamaterials, selective and sensitive sensing inside nanometer volumes has been demonstrated [6–8]. Moreover, even though THz waves have very low photon energy, the high strength of THz systems with the help of field enhancement of metamaterials additionally enables the induction of nonlinear phenomena such as ionization [9–11], high harmonic generation [12–14], electrical tunneling [15], and irreversible damage [16]. Furthermore, a metamaterial structure reconfigured by the electromigration of metals due to an intense THz field has been reported [15]. These effects provide a fundamental understanding of novel electronic structures and states of materials, discovering new physics of nonlinear THz-material interactions. Because an unexpected response may result in the permanent degradation of devices, such nonlinear effects governed by intense fields should be considered cautiously. Therefore, the threshold power that produces a nonlinear response should be determined. Electrical devices can be protected from permanent damage due to excessive current flow using an optical fuse placed in front of the core of a device.

Here, we demonstrate a THz optical nanofuse realized by irreversible modulations using nanowire-combined THz antenna structures. The nanofuse is composed of a semiconductor or metal nanowire placed on a THz nanoantenna, which is operated through the transmitted THz wave. We observed two different nanofuse modulations induced by the THz wave for the different nanowires: (1) the contacted semiconductor nanowire placed on the nanoantenna was disconnected, and (2) the floating metal nanowire placed on the nanoantenna was waved and touched the nanoantenna. Furthermore, after the metal-nanowire contact, it is degraded by the induced THz field, generating electromigration. Both nanowire modulations were enabled by the field enhancement of nanoantenna with an incident terahertz field of 20 kV/cm, which suggested a potential application as an optical fuse to protect sensitive terahertz devices from unavoidable damage or side effects.

## 2 Sample fabrication and experimental setup

**Figure 1:**
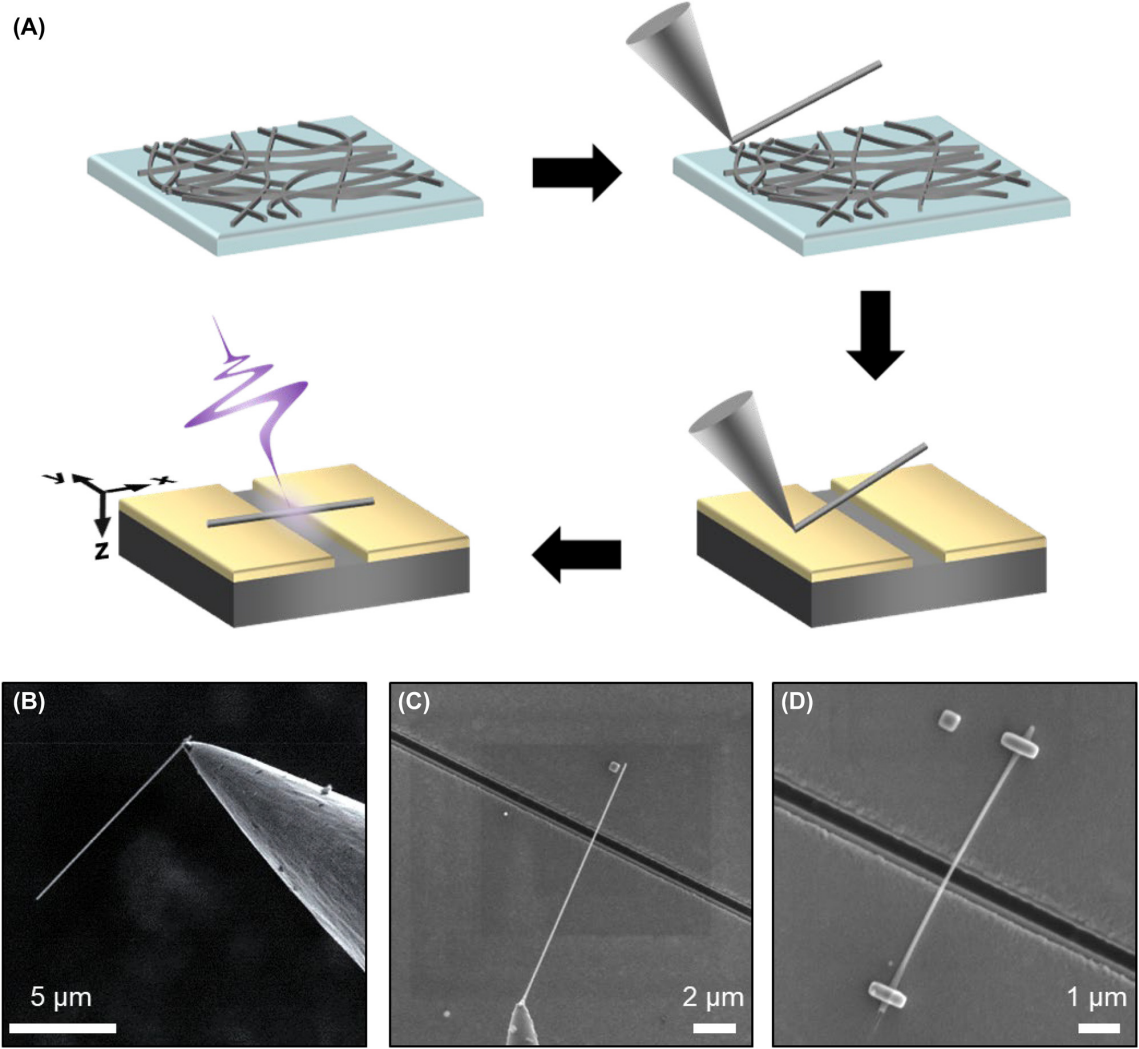
Fabrication of nanofuse systems. (A) Schematic of fabrication and measurement process of nanofuse antenna. (B–D) SEM images of fabrication of nanofuse antenna. (B) A single nanowire is attached to a sharp tungsten tip inside the SEM chamber. (C) The single nanowire is approached to the target place on gold nanoantenna, after then, (D) the nanowire is tightly bonded by platinum (Pt) deposited on the ends. Finally, rest of the nanowire is detached by the ion-milling process within the chamber.

Figure 1A shows the fabrication process of terahertz nanofuse with a nanowire. First, nanowires in solution were dispersed on the substrate, and inside a scanning electron microscopy (SEM) chamber, a single nanowire was attached to a sharp tungsten tip with a platinum source and controlled by a nano-manipulator (Figure 1B). The single nanowire approached the target location on the gold nanoantenna (Figure 1C). After the nanowire was approached, it was tightly bonded to platinum (Pt) deposited on the ends. Furthermore, the rest of the nanowires were detached using an ion-milling process within the chamber (Figure 1D). In Figure 1D, the length of the nanowire between the two Pt nodes is 5.3 μm, and the diameter of the nanowire is 82 nm. We fabricated a single nanoslot antenna with a width of 380 nm and length *l* of 120 μm on a 150 nm thick gold-coated silicon substrate using focused ion beam (FIB) lithography. The length of the nanoslot antenna was selected to set the fundamental resonant frequency at 0.43 THz, identified by the relation, $f_{\text{res}}=\frac{c}{\sqrt{2\left(n^{2}+1\right)}l}$ , where *c* is the speed of light and *n* is the real part of the refractive index of the substrate [16]. For the terahertz transmission experiment, an *x*-polarized THz wave impinged into the nanowire along the *z*-direction.

Terahertz time-domain spectroscopy was used for THz spectroscopic measurements. A regenerative amplifier laser pulse with pulse duration of 35 fs centered at a wavelength of 800 nm (repetition rate of 1 kHz and pulse energy of 3 mJ) was irradiated on a ZnTe crystal to generate intense THz radiation of ∼20 kV/cm. The generated THz wave was guided by a parabolic mirror and focused with a Tsurupica lens pair. The samples were placed at a 2 mm focal spot of the THz wave, oriented so that the polarization of the THz electric field was perpendicular to the long axis of the nanoslot antenna. This enabled us to excite optimally the resonant mode of the nanoslot antenna at a designed frequency while simultaneously inducing maximal absorption in the nanofuse systems. After transmission through the samples, the THz wave was guided by a Tsurupica lens and focused again onto the ZnTe crystal for detection using another parabolic mirror. Finally, the transmitted THz pulse was measured using electro-optic sampling. All measurements were performed at room temperature under weak vacuum surroundings (10^−2^–10^−3^ torr) and the transmitted field is through the 1 mm × 1 mm aperture, which means the transmitted area in our transmission experiment. The normalized transmittance, $T=\frac{{\left(E_{\text{sam}}\left(\omega \right)\right)}^{2}}{{\left(E_{\text{ref}}\left(\omega \right)\right)}^{2}}$ , was obtained by dividing the measured THz transmittance through each sample, ${\left(E_{\text{sam}}\left(\omega \right)\right)}^{2}$ , by the measured THz transmittance through a bare silicon substrate, ${\left(E_{\text{ref}}\left(\omega \right)\right)}^{2}$ .

## 3 Results and discussion

**Figure 2:**
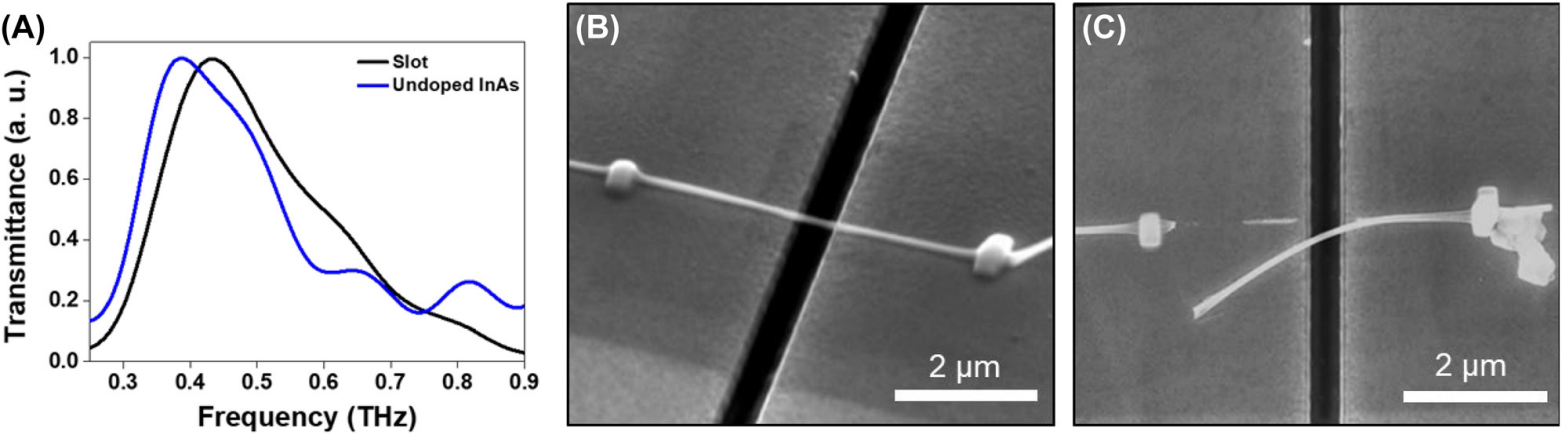
Semiconductor nanowire-based nanofuse systems and their spectral performance. (A) Normalized transmittance through a nanoantenna only, and a nanofuse with a nanowire located at the center position of the nanoantenna. The undoped InAs nanowire is used for the nanofuse antenna. (B–C) SEM images for the nanofuse (B) before and (C) after nanowire broken by the intense THz field.

The THz nanofuse experiments were designed as permanent reconfigurations of the designed systems in the opposite manner: breaking or creating nanowire bridges (nanobridges). The first type uses a well-defined nanowire bridge across a metal nanoslot structure. The THz field transmitted through a bare nanoslot antenna. Additionally, an undoped InAs nanowire located at the center position across the long axis of the slot antenna is shown in Figure 2B. Each measured THz transmission through the samples was normalized to the value obtained using a bare silicon substrate. Without the nanowire, the bare nanoslot antenna has a fundamental resonant frequency of 0.43 THz (corresponding to the length of the slot of 120 µm). The width of the nanoslot was 380 nm, resulting in a 250-fold THz field enhancement. This condition can be considered as the strongly enhanced field which can induce impact ionization by the additional acceleration of electrons supported by the ponderomotive force [17]. The fundamental resonance of the nanowire-bridged nanoantenna was changed to 0.38 THz (Figure 2A) [17, 18]. The resonance shift can be explained in terms of the refractive index of the nanowire. When the nanowire is placed on the confined THz field near the antenna gap, the confined field interacts with nanowire more effectively. As a result, effective refractive index of antenna increases by the change of the system’s optical properties, showing the red-shift of the resonance frequency. For the nanofuse experiment, a single nanobridge sample was exposed to the maximum THz field strength for 30 min. Figure 2B and C shows SEM images of the nanobridge before and after intense THz field exposure. Because of the intense THz field, the InAs nanowire bridge breaks, as shown in Figure 2C. This phenomenon is attributed to the highly enhanced field in the nanoslot antenna, which induces a nonlinear effect on the instant current flow through the nanowire. A large accumulation of charge carriers along the nanowire at the junction of one side of the Pt node causes an explosion over the critical field strength. Furthermore, the InAs nanowire bridge was finally broken because accelerated carriers at the abrupt interface between the semiconductor wire and Pt metal could not escape toward the gold metal plate in time and induced the collapse of the junction [19, 20].

**Figure 3:**
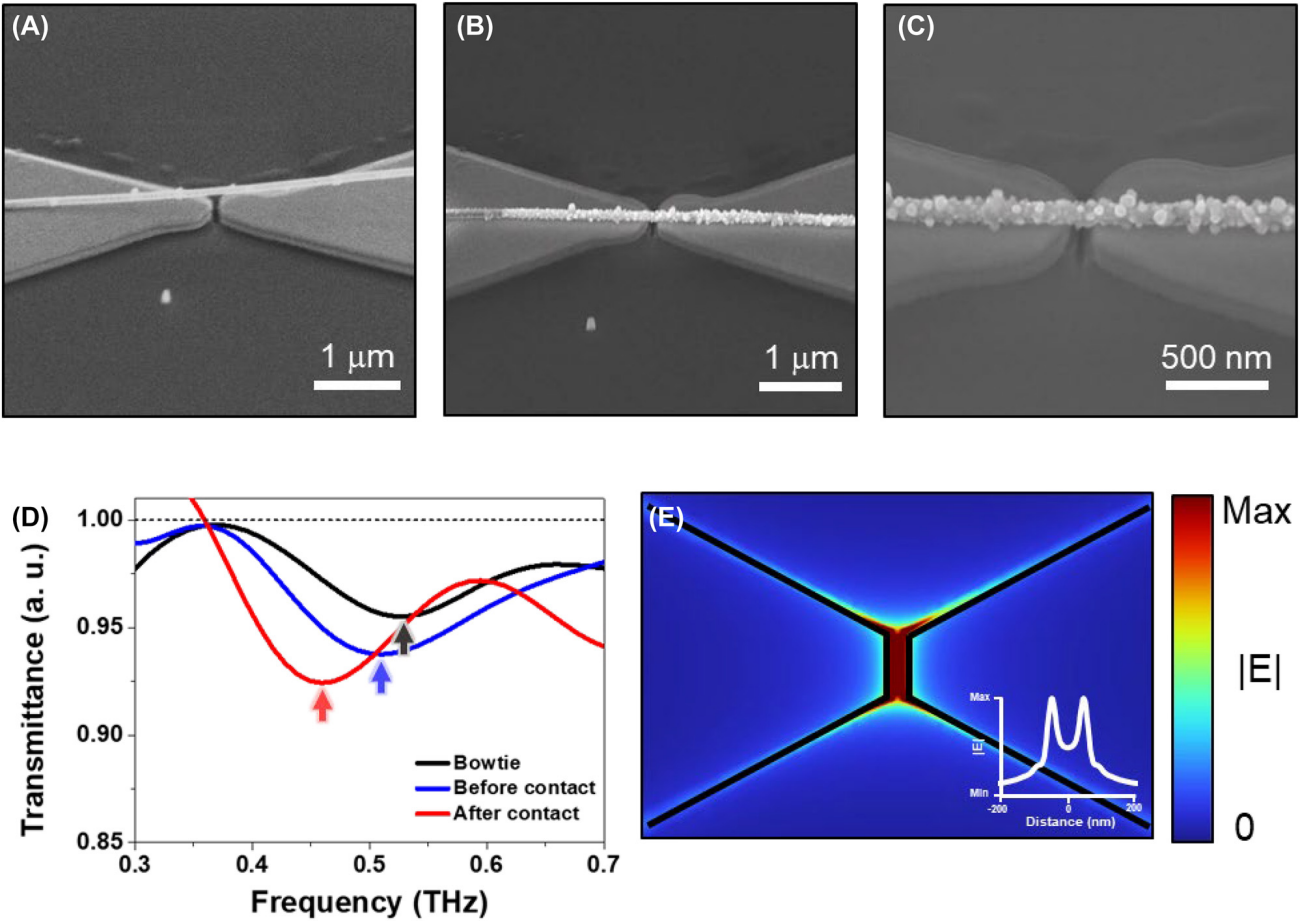
Metal nanowire-based nanofuse systems and the THz field distribution. SEM images of one-side fixed nanofuse device (A) before the nanowire contact and (B) after the nanowire contact. The single nanowire is placed on gold bowtie antenna. (C) SEM image of nanowire degradation by the induced terahertz field. (D) Normalized transmittance through a bare bowtie and nanofuse device. The resonant frequency of the bare bowtie and nanofuse device is 0.55 and 0.51 THz, respectively. After the nanowire contact, the resonant frequency of the nanofuse device is changed to 0.45 THz. (E) Terahertz electric field distribution near the bowtie antenna. The inset shows the electric field value between the bowtie gap.

To explore the influence of the high THz field on nanoscaled systems, another type of combination of nanowire and nanoantenna was investigated. A bowtie nanoantenna with metal nanowire was used for nanofuse. Owing to the narrower gap width of 90 nm and bowtie structures, a higher THz field enhancement can be obtained compared to a nanoslot antenna. The maximum field enhancement of the bowtie antenna is approximately 1600 at the gap edges, as shown in Figure 3E. In the case of the bowtie antenna-based nanofuse, a silver nanowire was selected for efficient charge transport, leading to strong movement of the bridge. The bowtie nanoantenna was fabricated using the electron beam lithography and FIB process. First, a diabolo-shaped antenna was fabricated on a highly resistive silicon substrate with a 50 nm gold layer by electron beam lithography, and afterwards, a 90 nm gap at the center of the diabolo was subsequently fabricated by FIB. Consequently, a bowtie was formed with dimensions of 100 × 80 μm and gap width of 90 nm. The bowtie antenna had a fundamental resonant frequency of 0.53 THz. After the fabrication of the bowtie nanoantenna, the silver nanowire of 85 nm in diameter and 6 μm in length was placed on one wing of the bowtie (Figure 3A), which changed the fundamental resonance of bowtie to 0.51 THz (Figure 3D).

To investigate the irreversible modulation of the nanofuse system, the nanobridge-bowtie was exposed to a maximum THz field strength for 30 min. After THz exposure, the silver nanowire met the other wing of the bowtie nanoantenna (Figure 3B) and then gradually degraded in terms of exposure time (Figure 3C). The mechanism involves two steps. First, the incident THz field is strongly confined in the gap of the bowtie nanoantenna and pulls the nanowire strongly, causes it to touch the surface of the bowtie. Perfectly contacting the nanowire to the surface created a nanobridge across the bowtie. Second, a high THz field deforms the nanobridge at the highest-field spot of the nanoantenna. As shown in Figure 3A, one side of a silver nanowire is attached to the bowtie, and then the THz field induces the nanowire to contact the other wing of the bowtie (Figure 3B). After contact, the confined THz field leads to the transition of nanowires, such as electromigration, as shown in Figure 3C. Electromigration occurred and transformed the silver nanowire completely. In the transmittance data, we observed a resonant frequency shift of 0.46 THz after nanowire contact (Figure 3D). Although the following frequency shift was not observed by electromigration of silver nanowires in the experiment, this suggests that the effective thickness of the nanowires or the intrinsic material properties did not change even after degradation. The bowtie resonance enhanced the THz field more than hundred times so that an electric field of ∼13 MV/cm at 0.45 THz, which was extracted from the field enhancement factor of 655 averaged across the nanogap.

The strong field strength enables electrons to overcome the work function barrier. Furthermore, the electrons traveling in the silver nanowire start escaping when a high voltage is applied. In the antenna structures, strong field confinement triggers electrically driven migration, i.e., electromigration, which has been reported previously [15, 21]. Owing to several elements such as impact heating and electron scattering momentum transfer, the edge lattice deforms and shows mass migration, constructing a new geometry. In our case, extremely small confinement and inhomogeneous field distribution stimulated mass transfer (Figure 3C). The transmittance spectra of each state of the nanowire are shown in Figure 3D, which show the different resonance behaviors. The change in the resonance frequency before and after nanowire contact implied that the nanowire position near the bowtie affected the resonance frequency. The highly confined electric field in the gap induced charges along the metallic nanowire, which caused an attractive force between the nanowire and the bowtie surface. The distance between the nanowire and bowtie decreased until the nanowire was attached to the bowtie surface, similar to nanofuse [22]. The nanowire on the bowtie formed a new (nanobridge) structure, which resulted in a frequency shift in the transmittance spectra.

**Figure 4:**
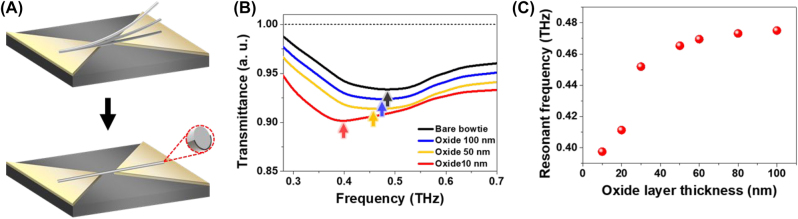
Simulations of the one-side fixed nanofuse device. (A) Effective modeling of one-side fixed nanofuse device. The one-side fixed nanofuse is considered as a nanowire placed on the bowtie nanoantenna with oxide spacing layer. (B) Calculated transmittance spectra of nanofuse device with various oxide layer thickness. (C) The resonant frequency for each spectrum along the thickness of the oxide layer is plotted.

To confirm the frequency variation of the nanofuse with floated nanowires (one-side fixed) from the experimental results, nanowires on the antenna spaced with metallic oxide layer was investigated by a COMSOL Multiphysics. The optical properties of the silver layer were determined by the Drude parameters, and the refractive index of silicon was *n* = 3.41. In the calculation, the metallic nanowire was spaced by an oxide layer between the bowtie antenna and nanowire, representing an effective model of the one-side fixed nanofuse. The thickness of the oxide layer is regarded as the angle between the bowtie antenna and silver nanowire. Moreover, as the angle between the bowtie nanoantenna and nanowire decreased, a thinner oxide layer was considered. The thickness of the oxide layer was varied from 100 to 10 nm. Here, the 10 nm oxide layer is represented by nanowire contact with the bowtie antenna. The transmittance spectra of the bare bowtie and three different oxide layers (100, 50, and 10 nm thickness) nanofuse samples are shown in Figure 4B. The resonance frequency of bowtie before the nanowire attachment is approximately 0.48 THz represented as a black line. After the nanowire was placed on the bowtie antenna with the oxide layer, the resonance frequency was red-shifted as the thickness of the oxide layer decreased. Figure 4C shows the resonant frequency of the nanofuse device obtained from the calculated results. When the oxide layer was decreased from 100 to 10 nm, the resonant frequency was red-shifted from 0.48 to 0.397 THz. The dramatic change in the resonant frequency from 30 to 10 nm is due to the effective field-confined volume [23]. From the frequency change, we can estimate the exact nanowire position for the proper operation of the nanofuse.

## 4 Conclusions

We demonstrated a THz optical nanofuse through irreversible modulations using nanowire-combined nanoantenna structures. We observed two different nanofuse modulations induced by enhanced THz waves. For semiconductor nanowire, the nanowire bridge across the metal-nano slot structure shows irreversible modulation by the nanowire broken, which is due to the heavy accumulation of charge carriers along the nanowire at the junction of one side of a Pt node. Furthermore, for the metal nanowire, we fabricated floated nanobridge-bowtie that nanowire was placed on one wing of a bowtie antenna. This nanobridge-bowtie moves the nanowire to contact the other wing of the bowtie antenna, and afterward it is degraded gradually by the enhanced THz field. In this case, the attractive force and electromigration effect result in irreversible modulation and degradation of the nanobridge-bowtie. We suggest that our irreversible modulation of nanobridge samples will open pathways toward optical fusion to protect sensitive THz devices from unavoidable damage or side effects.
